# Prognosis of West Nile virus associated acute flaccid paralysis: a case series

**DOI:** 10.1186/1752-1947-5-395

**Published:** 2011-08-19

**Authors:** Jennie Johnstone, Steven E Hanna, Lindsay E Nicolle, Michael A Drebot, Binod Neupane, James B Mahony, Mark B Loeb

**Affiliations:** 1 Department of Medicine, McMaster University, 1280 Main Street West, Hamilton, ON, L8S-4K1, Canada; 2 Michael G. DeGroote Institute for Infectious Disease Research, McMaster University, 1280 Main Street West, Hamilton, ON, L8S-4K1, Canada; 3 Department of Clinical Epidemiology and Biostatistics, McMaster University, 1280 Main Street West, Hamilton, ON, L8S-4K1, Canada; 4 Department of Internal Medicine, University of Manitoba, 820 Sherbrook Street, Winnipeg, MB, R3A-1R9, Canada; 5 Department of Medical Microbiology, University of Manitoba, 820 Sherbrook Street, Winnipeg, MB, R3A-1R9, Canada; 6 National Microbiology Laboratory, Health Canada, 1015 Arlington Street, Winnipeg, MB, R3E-3R2, Canada; 7 Department of Pathology and Molecular Medicine, McMaster University, 1280 Main Street West, Hamilton, ON, L8S-4K1, Canada

## Abstract

**Introduction:**

Little is known about the long-term health related quality of life outcomes in patients with West Nile virus associated acute flaccid paralysis. We describe the quality of life scores of seven patients with acute flaccid paralysis who presented to hospital between 2003 and 2006, and were followed for up to two years.

**Case presentations:**

Between 2003 and 2006, 157 symptomatic patients with West Nile virus were enrolled in a longitudinal cohort study of West Nile virus in Canada. Seven patients (4%) had acute flaccid paralysis. The first patient was a 55-year-old man who presented with left upper extremity weakness. The second patient was a 54-year-old man who presented with bilateral upper extremity weakness. The third patient was a 66-year-old woman who developed bilateral upper and lower extremity weakness. The fourth patient was a 67-year-old man who presented with right lower extremity weakness. The fifth patient was a 60-year-old woman who developed bilateral lower extremity weakness. The sixth patient was a 71-year-old man with a history of Parkinson's disease and acute onset bilateral lower extremity weakness. The seventh patient was a 52-year-old man who presented with right lower extremity weakness. All were Caucasian. Patients were followed for a mean of 1.1 years. At the end of follow-up the mean score on the Physical Component Summary of the Short-Form 36 scale had only slightly increased to 39. In contrast, mean score on the Mental Component Summary of the Short-Form 36 scale at the end of follow-up had normalized to 50.

**Conclusion:**

Despite the poor physical prognosis for patients with acute flaccid paralysis, the mental health outcomes are generally favorable.

## Introduction

In 1999, West Nile virus caused an outbreak in New York City and has since emerged as an important human pathogen in North America [1,2]. Although most cases of West Nile virus infection are asymptomatic, symptomatic disease can occur and ranges from a mild febrile illness (20% of infected individuals) to severe illness with central nervous system involvement (<1% of all infected cases) [3]. Classically, neurologic manifestations included meningitis and encephalitis; however, in 2002 the first cases of West Nile virus associated acute flaccid paralysis were described [4,5]. Since then, acute flaccid paralysis has become an established complication of West Nile virus, presumed to result from direct involvement of the anterior horn cells of the spinal cord by the infection [6].

The prognosis of patients with West Nile virus-associated acute flaccid paralysis is unclear. Several case series have evaluated physical recovery over time, and the majority of patients do not recover fully [6-11]. In one report, approximately one-third of the patients had a partial recovery, one-third recovered to near baseline levels, and one-third had almost no recovery [11]. The impact of the impaired physical function on quality of life has not been reported for cases of acute flaccid paralysis [12-14]. Improved understanding of the history of this disease, including its impact on health-related quality of life is necessary to provide information addressing prognosis for patients at the time of diagnosis.

We sought to describe the presenting features of seven cases of acute flaccid paralysis enrolled in a prospective cohort of 157 patients with symptomatic West Nile virus infection between 2003 and 2006 [13], and report their long term health-related quality of life outcomes. Acute flaccid paralysis was diagnosed if patients had a positive West Nile virus IgM antibody capture enzyme-linked immunosorbent assay confirmed by plaque reduction neutralization assay [2], associated with acute onset of limb weakness with marked progression over 48 hours and at least two of the following: asymmetric weakness; areflexia or hyporeflexia of affected limbs; absence of pain, paresthesia or numbness in affected limbs; cerebrospinal fluid pleocytosis and elevated protein levels; electrodiagnostic studies consistent with anterior horn cell process or abnormal increased signal in the anterior gray matter on imaging. Outcome measures included the Physical Component Summary (PCS) of the Short-Form-36, a standardized measure of physical function, and the Mental Component Summary (MCS) of the Short Form-36 scale and were measured at each visit [15,16]. The PCS and MCS scales correlate highly with gold standard measures of physical function (r = 0.85) and mental health (r = 0.87), respectively [16]. Patients were followed prospectively by trained research nurses and evaluated at pre-specified intervals (baseline (that is at the time of presentation), 30 days, six months, one year and two years after presentation). If a patient was lost to follow-up, the last recorded measure was used. For both scales, scores range from 0 - 100 with very high scores indicating a high level of function and very low scores indicating substantial impairment. All scores are standardized to the general US population using linear transformation (mean score 50, (SD, 10))[15,16].

## Case presentations

### Case one

A 55-year-old Caucasian man, with no known medical problems presented with acute onset of left arm weakness after a viral prodrome which included fever, fatigue, nausea, vomiting and headache (Table 1). On examination he was afebrile (temperature of 36.9°C), hemodynamically stable and had flaccid weakness of the left arm that extended from his left wrist, up to his left shoulder. Reflexes of the affected arm were absent. The remainder of the neurological examination was unremarkable. A lumbar puncture was not performed. The patient was admitted to the hospital for observation and further investigation. A contrast enhanced computed tomography (CT) scan of his neck was unremarkable. Serum IgM was positive for West Nile virus and the patient was diagnosed with West Nile virus acute flaccid paralysis affecting the left upper limb. He was discharged eight days later following an uncomplicated hospital stay. Once discharged, the patient underwent physiotherapy two times a week to help improve the strength in his left arm. The strength began to return two months following admission to the hospital, and six months later it had returned to baseline function. The PCS and MCS outcomes can be found in Table 2.

**Table 1:** Presenting clinical features of 7 cases of acute flaccid paralysis

	Baseline characteristics			Presenting clinical features					Therapeutic interventions		
**Case**	**Age**	**Sex**	**Co-morbidity**	**Fever**	**Involved sites**	**Intubation**	**CSF WBC (cells/μl)**	**CSF protein (g/L)**	**PT**	**OT**	**Antidepressants**

1	55y	M	None	No	LUE	No	-	-	Yes	No	No

2	54y	M	Hypertension Dyslipidemia	No	Bilateral UE	No	<5	<0.45	No	No	No

3	66y	F	None	No	Bilateral UE and LE	Yes	123	1.27	Yes	Yes	No

4	67y	M	None	No	RLE	No	189	1.04	Yes	No	No

5	60y	F	None	No	Bilateral LE	No	32	0.77	Yes	No	Yes (citalopram)

6	71y	M	Heart failure Parkinson's	Yes	Bilateral LE	No	<5	1.06	Yes	Yes	No

7	52y	M	None	No	RLE	No	39	1.35	Yes	No	No

Abbreviations: y, year; M, male; F, female; L, left; R, right; UE, upper extremity; LE, lower extremity; CSF, cerebrospinal fluid; WBC, white blood cell count; PT, physiotherapy; OT, occupational therapy.

**Table 2:** PCS and MCS outcomes

Case	PCS Scores						MCS Score					
	**Baseline**	**30-day**	**6 month**	**1 year**	**2 year**	**Change in score**^**†**^	**Baseline**	**30-day**	**6 month**	**1 year**	**2 year**	**Change in score**^**†**^

**1**	31	34	59	-	-	28	30	55	62	-	-	32

**2**	18	58	55	59	49	31	43	58	58	57	56	13

3*	-	-	-	28	-	-	-	-	-	33	-	-

**4**	45	29	41	42	-	-3	43	49	62	63	-	20

**5**	29	38	37	-	-	8	33	39	34	-	-	1

**6**	57	39	28	24	24	-33	12	22	37	53	50	38

**7**	26	32	36	-	-	10	41	55	54	-	-	13

*The first three measurements were not obtained as the patient was ventilated and sedated in ICU.

^†^Score at the end of follow-up minus the score at baseline.

### Case two

A 54-year-old Caucasian man with a history of hypertension and dyslipidemia developed a viral prodrome of fatigue, rash, nausea, vomiting, diarrhea and a low grade headache (Table 1). He presented with coffee ground emesis to the hospital. In the emergency room he was febrile (temperature of 39.1°C) but hemodynamically stable. Neurological examination revealed mild left leg weakness but he was otherwise normal. He was admitted to the hospital for rehydration and was discharged two days later. West Nile virus serum IgM performed on admission was positive. Four days after discharge, the patient was re-admitted for investigation of progressive left leg weakness. He was afebrile (temperature 36.1°C). Neurological examination revealed markedly reduced strength in his left hip and quadriceps and absent left knee and ankle reflexes. Sensation was intact in his left lower limb and the remainder of the physical examination was unremarkable. Lumbar puncture was normal (<5 white blood cells/μL and protein <0.45 g/L in the cerebrospinal fluid (CSF)). Electromyography (EMG) findings were in keeping with peripheral nerve demyelination with some element of neuronal injury. A diagnosis of West Nile virus acute flaccid paralysis was made and he was discharged home 24 hours later; by the time of discharge the left leg weakness had progressed to include foot drop. Over the first few months following discharge the left hip and quadriceps weakness improved but the foot drop persisted. The patient still had persistent foot drop at the end of the study. The PCS and MCS outcomes can be found in Table 2.

### Case three

A 66-year-old Caucasian woman with no known medical problems was admitted with a two-day history of fever, headache, neck stiffness and photophobia (Table 1). She was brought to the hospital when she developed rapidly progressive bilateral upper and lower extremity weakness. In the emergency room her temperature was 37.7°C, her pulse was 98 beats/minute and her blood pressure was 180/78 mmHg. She was intubated to protect her airway and she had complete flaccid paralysis of bilateral upper and lower extremities. Lumbar puncture revealed 123 white blood cells/μL and protein of 1.27 g/L in the CSF. Serum IgM was positive for West Nile virus and a diagnosis of West Nile virus acute flaccid paralysis was made. While in the intensive care unit she was unable to be weaned from the ventilator due to ongoing weakness and required a tracheostomy. After one year, she had some improvement in strength in her limbs but required ongoing ventilatory support. The PCS and MCS outcomes can be found in Table 2.

### Case four

A 67-year-old previously healthy Caucasian man with acute onset right lower leg weakness one week following a viral prodrome of fever, headache, myalgias and photophobia (Table 1) presented to the hospital. In the emergency room he was afebrile (temperature of 36.5°C) and hemodynamically stable. Neurological examination revealed flaccid paralysis of the right leg and absent patellar and ankle reflexes. The remainder of his neurological examination was normal. Lumbar puncture was abnormal with 189 white blood cells/μL and 1.0 g/L of protein in the CSF. He was admitted to the hospital for observation and further examination. Serum IgM was positive for West Nile virus and an EMG was consistent with West Nile virus acute flaccid paralysis. He was discharged home after a 14-day stay. Once discharged, the patient underwent regular physiotherapy to help improve the strength in his leg. Although the strength improved with time, he continued to have significant weakness at the end of follow-up. The PCS and MCS outcomes can be found in Table 2.

### Case five

A 60-year-old previously well Caucasian woman with acute onset bilateral lower extremity weakness after a viral prodrome which included headache, fatigue, malaise and myalgias presented to the hospital (Table 1). Physical examination of the lower extremities revealed marked weakness of the right hip flexors, mild weakness of the left hip flexors with better distal strength bilaterally. Patellar reflexes were absent bilaterally but the ankle reflexes were intact. Sensation was normal and the remainder of the neurological examination was normal. A lumbar puncture was abnormal with 32 white blood cells/μL and 0.77 g/L of protein in the CSF. The patient was admitted for observation and further examination. An MRI scan showed diffuse enhancement of the cauda equina and nerve roots of the lumbosacral spine. Serum IgM was positive for West Nile virus and EMG was consistent with multiple root inflammation of the cauda equina, thus a diagnosis of West Nile virus acute flaccid paralysis was made. The patient was discharged home after 10 days in the hospital. Following discharge she received physiotherapy. Her strength slowly improved although at the end of follow-up she still required a walker for ambulation. Her PCS and MCS outcomes can be found in Table 2.

### Case six

A 71-year-old Caucasian man with a known history of heart failure and Parkinson's disease with rapidly progressive bilateral lower extremity weakness following a viral prodrome of fever, rash, neck stiffness, fatigue and myalgias (Table 1) presented to the hospital. In the emergency room he was febrile (temperature of 39.7°C) but hemodynamically stable. He was not oriented to time or place. He had flaccid paralysis of his lower extremities bilaterally and absent patellar and ankle reflexes. Neurological examination of his upper extremities showed increased tone and tremor consistent with the diagnosis of Parkinson's disease. Lumbar puncture results were abnormal; there were no white blood cells (<5 cells/μL) in the CSF but the protein was elevated at 1.06 g/L. He was admitted to the hospital for observation and further examination. Serum IgM was positive for West Nile virus and a diagnosis of West Nile virus acute flaccid paralysis was made. The delirium quickly cleared and after one week in the hospital, his strength began to return. After one month in the hospital his strength continued to improve but was not at baseline, thus he was transferred to an in-patient rehabilitation facility. Although his strength ultimately returned to baseline, his overall mobility declined with time due to his Parkinson's disease. His PCS and MCS outcomes can be found in Table 2.

### Case seven

A 52-year-old Caucasian man with no known medical problems presented with acute onset of right leg weakness following a viral prodrome of fever, headache, neck stiffness, nausea, vomiting, myalgia and fatigue (Table 1). On examination he was afebrile (temperature of 35.9°C), hemodynamically stable and had flaccid weakness of the entire right lower leg. Reflexes of the affected knee and ankle were absent. The remainder of the neurological examination was unremarkable. A lumbar puncture was abnormal with 39 white blood cells/μL and 1.35 g/L protein in the CSF. He was admitted to the hospital for observation and further examination. An MRI scan of the spine was unremarkable. An EMG revealed denervation in the right adductor longus muscle. Serum IgM was positive for West Nile virus and the patient was diagnosed with West Nile virus acute flaccid paralysis affecting the right lower limb. He was discharged nine days later following an uncomplicated hospital stay. Once discharged, he underwent physiotherapy two times a week. His strength began to return 10 days following admission to the hospital, and six months later it had almost returned to his baseline function. The PCS and MCS outcomes can be found in Table 2.

## Summary of Cases

The mean age of those with acute flaccid paralysis was 61 years [SD, 7]. Most patients with acute flaccid paralysis were men (71%) and all were Caucasian. Almost one-third (28%) of patients with acute flaccid paralysis had any underlying co-morbidity. All patients with acute flaccid paralysis presented with acute onset, within 48 hours, of extremity weakness. A single extremity was involved in three patients and three patients had bilateral upper or lower limb involvement. One patient had complete flaccid paralysis of both upper and lower extremities; this patient required intubation and prolonged intensive care unit admission because of respiratory failure. No other patients required an intensive care unit admission. Length of hospital stay in an acute care facility ranged from 0 to 333 days (median 11 days). No patient died during the follow-up period.

Patients with acute flaccid paralysis were followed for a mean of 1.1 years [SD, 0.68]. The PCS and MCS scores of patients with acute flaccid paralysis from each visit can be found in Table 2. The mean PCS score at presentation was 34 [SD, 14] and the mean MCS score was 34 [SD, 12]. At the end of follow-up, the mean PCS score had only slightly increased to 39 [SD, 12] whereas the mean MCS score had normalized to 50 [SD, 12]. Patient number six appeared to be an outlier as his PCS score decreased over time; this might be explained by his history of Parkinson's disease which could confound the results. As a sensitivity analysis, the mean PCS and MCS scores were recalculated without Patient number six's data and the scores were as follows: the mean PCS score at baseline was 30 [SD, 10] and increased to 42 [SD, 11] at the end of follow-up whereas the mean MCS score at baseline was 38 [SD, 6] and increased to 50 [SD, 13] at the end of follow-up.

The change in score over time was also calculated (Table 2). A change score was only possible for six patients as a baseline score was not available for Patient number three. The mean PCS change score was 7 [SD, 23] and the mean MCS change score was 20 [SD, 14]. When the scores for Patient number six were excluded from the analysis, the mean PCS change score was 15 [SD, 14] and the mean MCS change score was 16 [SD, 11].

## Discussion

Acute flaccid paralysis affected 4% of all subjects with infection in this cohort, and most cases occurred in healthy adults. The incidence of West Nile virus acute flaccid paralysis in the general population has been estimated at four out of 100,000 during epidemics [9]. Neuroinvasive manifestations are thought to occur in <1% of all West Nile virus infections [3] and acute flaccid paralysis is thought to cause 5% to 10% of all cases of neuroinvasive disease [9]. Clear risk factors for acute flaccid paralysis have not been reported. Contrary to other forms of West Nile virus neuroinvasive disease, most cases of acute flaccid paralysis occur in healthy, non-elderly individuals [9,17,18], although advanced age may be associated with an increased risk of mortality [6]. In our case series, five of the seven patients with acute flaccid paralysis had no known co-morbidity and the mean age was less than 65 years, reinforcing previous observations.

After a mean follow-up of 1.1 years, the physical recovery was poor; however the mental health outcomes appeared to be generally favorable. The poor physical outcome seen in patients with acute flaccid paralysis is consistent with the incomplete physical recovery seen in this patient population [6,9]. Persistent deficits reflect the pathophysiology, as the damage to the anterior horn cells of the spinal cord appears to be irreversible [6]. The relative recovery of mental health outcomes was unexpected. Normalization of the MCS scores for West Nile fever and meningoencephalitis has been seen in a previous study [12], but to the best of our knowledge, this has not been documented in patients with acute flaccid paralysis. The relative recovery of mental health outcomes is encouraging and may help when discussing prognosis.

The patient with Parkinson's disease may have confounded the results and led to an underestimate of improvement in mean PCS scores, but in a sensitivity analysis the exclusion of this patient had little effect on the MCS scores. In addition, we could not rule out the possibility that some of the patients in this case series also had concomitant non-severe encephalitis which could bias the results towards underestimating improvement.

## Conclusion

Acute flaccid paralysis is an uncommon but serious manifestation of West Nile virus infection. It should be suspected in any individual, regardless of age or co-morbidity, who presents with weakness following potential exposure to mosquitoes. Most individuals will not recover full physical function, but mental health outcomes appear to recover and are comparable to those seen with other forms of symptomatic West Nile virus infection. Although this study is limited by the small number of cases, there is currently a paucity of data describing the long-term quality of life outcomes of this rare disease, and these hypothesis-generating results provide a foundation for future studies designed to describe the prognosis of patients with West Nile virus associated acute flaccid paralysis.

## Consent

Written informed consent was obtained from all patients for publication of these case reports and any accompanying images. Copies of the written consents are available for review by the Editor-in-Chief of this journal.

## Competing interests

The authors declare that they have no competing interests.

## Authors' contributions

JJ drafted the manuscript. SH participated in its design and helped draft the initial manuscript. LN helped conceive the study and design, helped coordinate the study and critically revised the manuscript. MD performed the West Nile virus testing and critically revised the manuscript. BN critically revised the manuscript. JM performed West Nile virus testing and critically revised the manuscript. ML conceived the study and design, acquired the data, critically revised the manuscript and gave final approval of the version to be published.
